# A Case of Salmonella Dublin Empyema Associated With Refractory Ulcerative Colitis and Colorectal Carcinoma

**DOI:** 10.7759/cureus.86480

**Published:** 2025-06-21

**Authors:** Mohamed M Elfeky, Abdelrahman A Mohamed

**Affiliations:** 1 Internal Medicine, Prince Philip Hospital, Hywel Dda University Health Board, Llanelli, GBR; 2 Respiratory Medicine, Glangwilli Hospial, Hywel Dda University Health Board, Carmarthen, GBR

**Keywords:** colorectal cancer, empyema, immunosuppression, salmonella dublin, ulcerative colitis

## Abstract

Non-typhi *Salmonella* infection rarely presents as *Salmonella* empyema. We report a case of a 71-year-old male with worsening left-sided pleuritic chest pain. A chest radiograph revealed a left-sided pleural effusion, and pleural fluid culture grew *Salmonella *Dublin. This occurred in the context of refractory ulcerative colitis and a subsequent diagnosis of colorectal carcinoma.

## Introduction

*Salmonella* is a motile Gram-negative bacillus first described by Salmon and Smith in the 1880s [1]. *Salmonella* most commonly presents as gastroenteritis and enteric fever. Non-typhi *Salmonella* are often associated with foodborne illness, but they can also cause systemic infections, especially in immunocompromised patients. The occurrence of pleuropulmonary disease caused by non-typhi *Salmonella* is rare. Only about 31 cases of *Salmonella*-related pleural infections have been reported over the past century [2]. Here, we report a case of *Salmonella* Dublin empyema in a patient with a background of resistant ulcerative colitis with a subsequent diagnosis of localized colorectal carcinoma. This case highlighted the importance of recognizing atypical infections that cause empyema in immunocompromised patients and those with chronic illnesses, as well as the need for early and proper management and identifying the source of infection.

## Case presentation

A 71-year-old male farmer was admitted to the hospital with left-sided pleuritic chest pain. He had been well and actively farming until 10 days prior, when he had this pain. He denied any other symptoms, including fever, rigors, cough, or shortness of breath.

There was a long history of refractory ulcerative colitis despite trials of azathioprine and infliximab, and surgery was recommended, which the patient had declined. His other comorbidities included diabetes mellitus, coronary artery disease, and prostate cancer, which was in remission.

On admission, his temperature was 38.1°C, respiration rate 32 breaths/min, pulse rate 78/min, and blood pressure 118/75 mmHg. On examination, there were decreased breath sounds and coarse crepitations over the left posterior chest but no tenderness or other relevant signs. Significant laboratory findings on admission are summarized in Table 1.

**Table 1:** Significant laboratory findings on admission

Test	Result	Reference range	Units
White blood cell count	13,500	4,000-11,000	cells/mm³
Neutrophil count	11,800	2,500-7,500	cells/mm³
C-reactive protein	185	<5	mg/L
Serum sodium level	128	135-145	mEq/L

**Figure 1:**
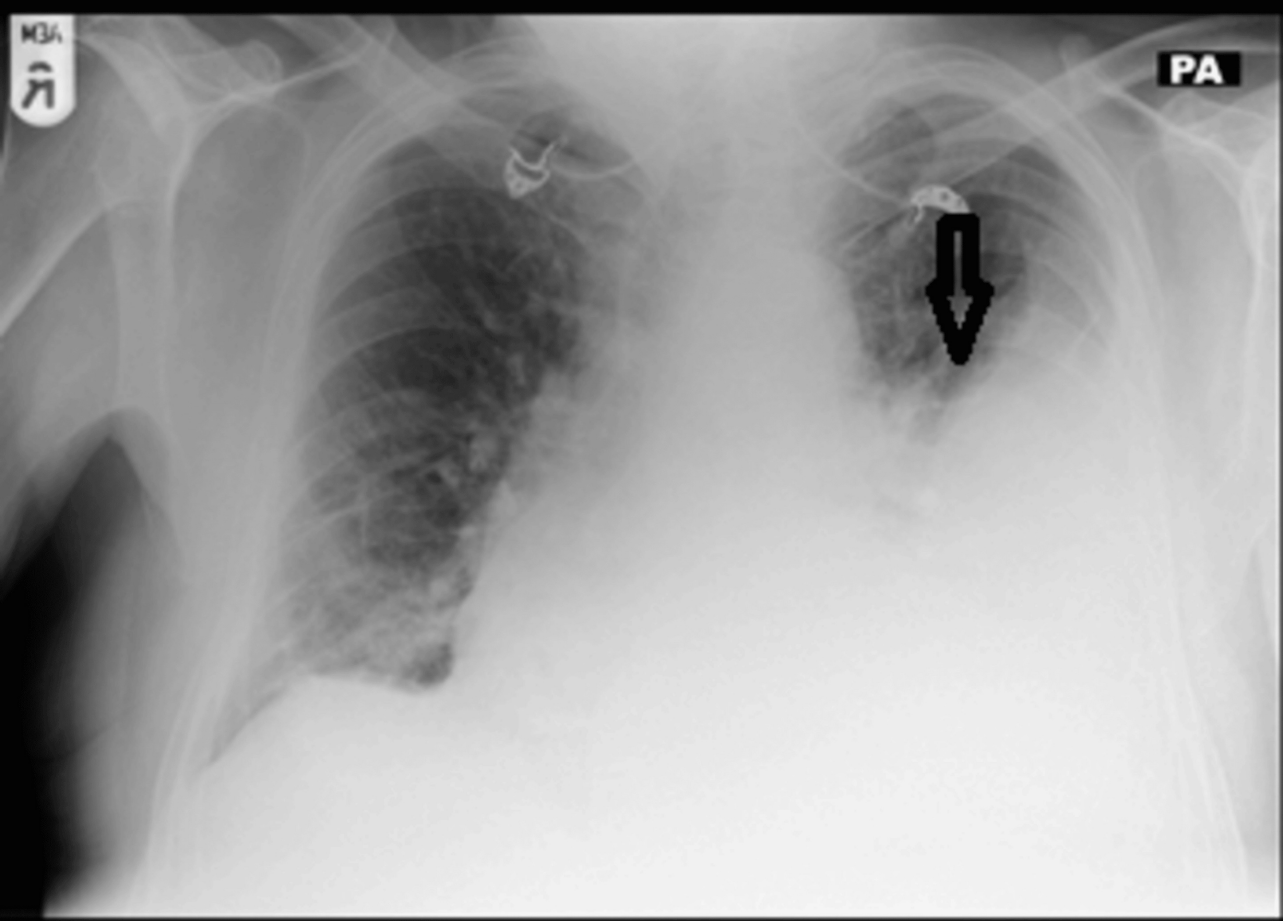
Admission posteroanterior view chest X-ray showing left pleural effusion

An admission chest X-ray revealed a large left-sided pleural effusion with opacification of half of the left hemothorax (Figure 1). Blood, stool, and urine samples were taken for routine cultures. The patient was diagnosed with left community-acquired pneumonia with a parapneumonic effusion and was commenced on meropenem as per local antibiotic guidelines. Additionally, azathioprine, which he was taking to control the refractory ulcerative colitis, was discontinued due to the ongoing infection.

The patient underwent a diagnostic left pleural aspiration, which revealed turbid pleural fluid. Biochemical analysis demonstrated a pH less than 7.2, a markedly low glucose level of 0.2 mmol/L, and a high protein level of 62 g/L, findings consistent with an exudative pleural effusion. Furthermore, pleural fluid culture grew *Salmonella* Dublin (Table 2).

**Table 2:** Pleural fluid analysis pH: potential of hydrogen, LDH: lactate dehydrogenase

Test	Result	Reference range	Units
Appearance	Turbid	-	-
pH	Unrecordable	>7.2	-
Glucose	0.2	>3.3	mmol/L
Protein	62	<30 (transudate)/>30 (exudate)	g/L
LDH	<10	-	U/L
Pleural fluid culture	*Salmonella* Dublin	-	-

**Figure 2:**
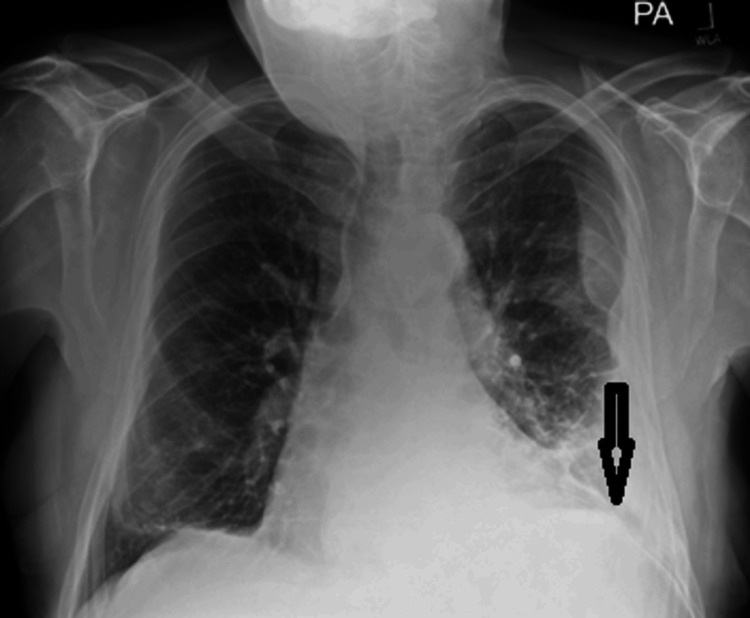
Post drainage posteroanterior view chest X-ray showing partial resolution of empyema, chest drain in-situ and left lower zone pulmonary infiltrates

Multiple blood cultures taken during his first 48 hours of hospital admission, whenever he spiked a temperature, showed negative results with no significant growth. Repeated urine and stool cultures were also negative. Similarly, his immunoglobulin levels, hepatitis serology, and HIV tests were normal. A total of 3.8 L of pleural fluid was drained via the chest drain (Figure 2), and he had improved both clinically and biochemically on meropenem. Eventually, his WBC and CRP normalized, and the chest drain was removed on day 12.

**Figure 3:**
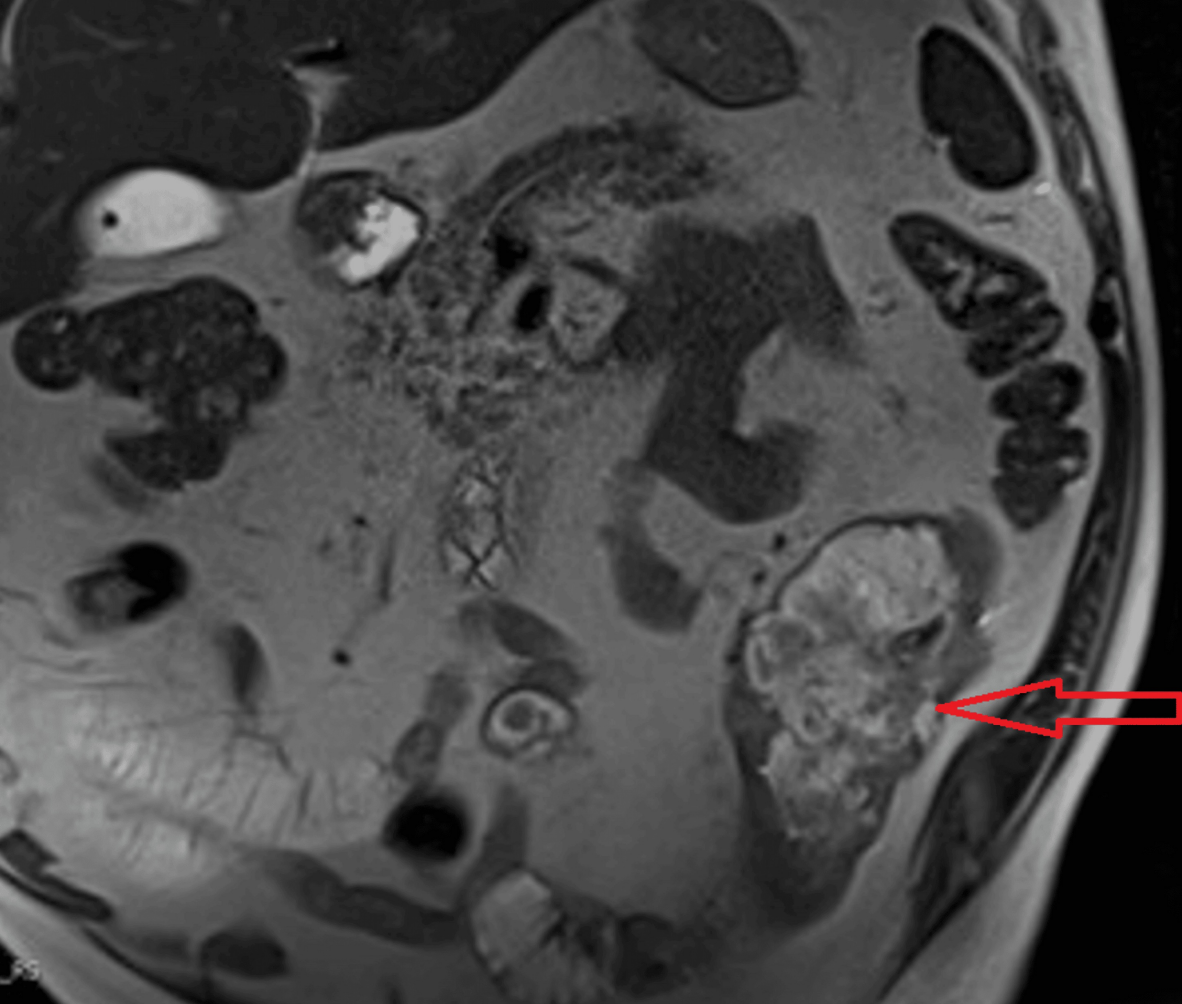
MRI T2 coronal view showing wall thickening and edematous mesentery involving descending colon MRI: magnetic resonance imaging

Based on guidance from the microbiologist, he was switched to ciprofloxacin after completing 14 days of meropenem. He then completed a total of eight weeks of antibiotics without recurrence of infection or reaccumulation of the left pleural effusion. Due to his history of refractory ulcerative colitis and ongoing diarrhea, he underwent an MRI of the thorax, abdomen, and pelvis, which showed extensive wall thickening and luminal distortion involving 12cm of the descending colon (Figure 3). A colo-pleural fistula was not identified.

Following discharge, he underwent a colonoscopy and biopsy of his descending colon, which demonstrated cancerous features, and he was diagnosed with T4N1M0 left colorectal carcinoma. He subsequently had a hemicolectomy and was referred to the oncology team for further management.

## Discussion

*Salmonella* infection commonly causes gastroenteritis and enteric fever. Pleuropulmonary infection with non-typhi *Salmonella* is rare. Only about 39 cases have been reported over the last century [1]. Risk factors for non-typhi *Salmonella* empyema include immunocompromised patients, such as those with hematological disease [3], type 2 diabetes mellitus [4], HIV infection, and tuberculosis [5]. Other risk factors include pre-existing lung pathology [6].

Non-typhi *Salmonella* can spread to the pleura through several routes. It can be through direct spread from a nearby infection like splenic abscess [7] or through hematogenous spread from the gastrointestinal tract to the pleura [8-10]. Therefore, isolation of enteric organisms from the pleural fluid should encourage clinicians to exclude any intra-abdominal source of infection.

Symptoms are often present with acute respiratory complaints [1]. A complete blood count may show leukocytosis [9]. Blood culture can be positive for *Salmonella* [3] or negative [2] due to low bacterial load. Pleural fluid analysis, including culture and sensitivity, is the key to diagnosing *Salmonella* pleural infection [9]. Chest radiographs usually show signs of pleural effusion [2].

Treatment of *Salmonella* empyema typically involves an extended course of antibiotics, including beta-lactams (e.g., ceftriaxone) or fluoroquinolones, which offer improved lung penetration [1]. Resistance to antibiotics can occur despite adequate antimicrobial therapy [11]. Pleural drainage for empyema and fibrinolysis are essential in the management [5]. Surgical intervention with decortication is necessary in complicated cases that show limited improvement with antibiotics, drainage, and pleurodesis [11].

## Conclusions

This case concluded a rare *Salmonella* Dublin empyema infection. This was associated with an immunocompromised state in the form of refractory ulcerative colitis and immunosuppressive medications. It also highlights the importance of tracing the source of infection, particularly the gastrointestinal source. Early diagnosis and management, including antibiotics and drainage, are the core components of treatment. Further investigation of the ongoing symptoms has led to the early diagnosis of colorectal cancer with proper management.
